# Notes on Preparations for the Sick

**Published:** 1912-06

**Authors:** 


### Notes on Preparations for the Sick.

**"Collosol" Argentum and "Collosol" Hydrargyrum.**—Oppenheimer & Sons, Queen Victoria Street, London.—These are colloidal solutions of the two metals mentioned. Such solutions are being introduced for therapeutic uses, and possess many advantages over dissolved metallic salts. Since our previous report (*vide* page 85) we have made a chemical examination of these preparations, and find that the solutions fail to give "ionic" reactions, *e.g.* they are not precipitated by addition of potassium iodide, showing absence of metallic salts.

Hydrogen sulphide causes a separation of the metal, which is a general reaction with solutions of this type.

**Trommer Elixir of Cod Liver Oil Extractives with Liquid Malt.**—Trommer Co. Ltd., 27 Charterhouse Square, London, E.C.—This preparation is stated to contain the proximate principles of Cod Livers and the finest Malted Barley in combination with Glycerophosphates. The makers claim that it possesses all the advantages of Pure Cod Liver Oil.

An examination shows the elixir to be miscible with water, and to possess a very agreeable taste. Considerable amounts of phosphorus are present in the ash. As an elegant malt product the extract leaves nothing to be desired, but in our opinion nothing can take the place of cod liver oil itself, administered in some form where its inherent properties are unaffected.

---

**Guttaplasts, Leucoplast, Nivea Soap, Eucerinum.**—P. BEIERSDORF & Co., Hamburg.—Samples of these excellent preparations are before us.

A long list of medicated plasters, made in accordance with the directions of Prof. Unna, stating their contents in grammes, and spread on a thin and flexible gutta-percha, combined with muslin; these are called Guttaplasts, or Beiersdorf's Gutta-percha Plaster Mulls.

Leukoplast is a rubber plaster of superior type of great pliability and durability, non-irritating, its whiteness being due to zinc oxide, which gives the adhesive plaster its colour and its name.

Nivea is a super-fatted soap, with many medicated variations, perfumed or otherwise.

Eucerinum anhydricum is an ointment base prepared from the pure wax alcohols of wool fat. The Eucerinum c. Aqua is preferable for ointments or pastes which are to be prepared with powders insoluble in water.

---

**Ureabromin—Bromide of Calcium-Carbamide.**— KNOLL & Co., Harp Lane, London.—This crystalline combination, easily soluble in water, and containing 36 per cent. of bromine, has given excellent results in the "Status Epilepticus." It combines the well-known action of bromine with the diuretic properties of urea and the cardiotonic effect of calcium. It has a minimum of toxicity, especially as regards bromide acne, and is put up in the convenient form of 15 grain tablets. It will need a very prolonged experience before one could say that the combination is superior to others containing the same bromine equivalent.

---

**Digipuratum.**—KNOLL & Co., Harp Lane, London.—A note on this standardised preparation appeared in vol. xxvii, p. 86. We have now received some sample ampoules of the fluid preparation, which is faintly alkaline and isotonic with the body fluids and intended for hypodermic injection. Each ampoule contains 1 c.c., representing 8 frog units of pharmacological activity. A single dose of 1 c.c. may be repeated four times daily. When hypodermic use of digitalis is required this preparation, always ready, is likely to be most convenient and efficient.

**Pond's Plasters.**—JOHN MORGAN RICHARDS & SONS, London.—Pond's Tampons are well known and much appreciated. The Plasters are practically a medicated glycerine poultice, being composed of a medicated glycerine and gelatine compound, filling the meshes of the cotton and gauze, and a waterproof backing makes the plaster a cleanly but soft and pliable application. Three varieties are made :—

(*a*) Pond's Pink Plaster, containing methyl salicylate and boric acid.

(*b*) „ Ichthyol Plaster, containing 5 per cent. of Ichthyol.

(*c*) „ Thigenol Plaster, with thigenol, boric acid, and tincture of iodine.

The multifarious uses of such plasters are obvious.

---

**Iodex c Methyl Salicylat.**—MENLEY & JAMES, London.—In vol. xxix we drew attention to an ointment called Iodex, containing resublimed iodine in a neutral base. We have now received a combination with Methyl Salicylate, which should be most useful in cases of chronic arthritis when the analgesic qualities of the latter drug are added to the iodine base. It should have a large range of utility.

---

**Zematone — Anti-asthmatic powder and cigarettes.**—ZEMATONE CO., Nottingham.—Yet another specific powder for inhalation in asthma. Its composition is elaborate, and it is made up in two strengths, labelled No. 1, the stronger ; No. 2, the weaker. It contains the organic bases used in such powders—stramonium, belladonna, red poppy, grindelia and hyoscyamus, with agaricin, sodium nitrate and potassium nitrate. It lights up easily, scintillates well, and gives off the fumes which commonly give so much relief when inhaled by asthmatics. The cigarettes are well made with a mouthpiece, and are not unpleasant to smoke.

---

**Dental Hypodermic Syringe.**—BURROUGHS, WELLCOME & CO., London.—Messrs. Burroughs, Wellcome & Co. have recently issued an improved pattern of their Dental Hypodermic Syringe. With the improvements now made it may be regarded as the ideal dental syringe. Its characteristics are summed up in the three words—strength, simplicity and asepticity. It is very strongly made in metal throughout, and is easily and rapidly put together for use and taken apart for cleaning and sterilisation. In mounting the syringe, the barrel, cap, nozzle and needle attachment are locked together by a simple arrangement, which does away with the use of screwthreads, washers and other parts, easily damaged or lost.

In the new syringe this locking device has been much strengthened. Every part being of metal, the syringe can be readily rendered sterile, and for this purpose the metal case in which it is supplied may be used as a sterilising tray. The Burroughs, Wellcome & Co.'s Dental Hypodermic Syringe takes the convenient unmounted needles now so largely used. An ingenious finger-grip, which can be fixed at any point on the barrel, and a screw to regulate the amount of fluid injected, give the final touches of detailed completeness to this extremely well-contrived and well-executed appliance.

